# Effect of intermittent theta-burst stimulation on chronobiological hypothalamic-pituitary-thyroid axis activity in resistant depressed patients

**DOI:** 10.1192/j.eurpsy.2025.888

**Published:** 2025-08-26

**Authors:** F. Duval, M.-C. Mokrani, V. Danila, T. Weiss, F. Gonzalez Lopera, M. I. Tomsa

**Affiliations:** 1 Psychiatry, Centre Hospitalier, Rouffach, France

## Abstract

**Introduction:**

So far, the effects of intermittent theta-burst stimulation (iTBS) treatment—a form of repetitive transcranial magnetic stimulation (rTMS) technique—on the hypothalamic-pituitary-thyroid (HPT) axis activity are poorly understood. In depression, and especially in treatment resistant depressed patients (TRDs), this axis is often dysregulated. We have previously demonstrated that the difference between the 23:00 h and 08:00 h thyrotropin (TSH) response to protirelin (TRH) tests on the same day (∆∆TSH test) is a very sensitive chronobiological index since it is reduced in about three quarters of major depressed inpatients.

**Objectives:**

The present study aimed at assessing the effects of iTBS treatment applied to the left dorsolateral prefrontal cortex (LDPFC) in hospitalized TRDs (defined as having at least 2 treatment failures) with abnormal chronobiological HPT functioning at baseline (BL).

**Methods:**

The ∆∆TSH test was performed in 18 TRDs and 18 matched healthy hospitalized control subjects (HCs). To be enrolled in this study, patients had to show at BL reduced ∆∆TSH values (i.e., < 2.5 mU/L) and a score of 18 or greater on the 17-item Hamilton Rating Scale for Depression (HAMD-17). All included TRDs were treated with antidepressants at the time of hospital admission. Drug dosages remained unchanged over the past month and kept stable throughout the course of iTBS. The ∆∆TSH test was repeated in all inpatients after 20 iTBS sessions (single daily session for 5 days of the week). Clinical response was defined as a reduction in HAMD-17 total score > 50% from BL and a final HAMD-17 score ≤ 8.

**Results:**

Compared to HCs, ∆∆TSH values were lower in TRDs at BL (p < 0.00001 by U test). After 20 iTBS sessions, HAM-D scores decreased (p = 0.001 by T-test) and ∆∆ TSH values increased (p = 0.01 by T-test) compared to BL, although endpoint ∆∆TSH values remained lower than those of HCs (p = 0.02 by T-test). However, there was a relationship between the reduction in HAM-D scores from BL to endpoint and the increase in ∆∆TSH values (rho = - 0.54; n = 18; p = 0.02). At endpoint, 10 patients (55%) showed ∆∆TSH normalization (among them 8 [80%] were responders), while 8 patients (45%) did not normalize their ∆∆TSH (all were non-remitters) (p = 0.001 by Fisher Exact test).

**Conclusions:**

Although the underlying mechanisms remain to be elucidated, the results of our present pilot study in TRDs suggest that successful iTBS treatment can restore a normal chronobiological activity of the HPT axis and vice versa.

**Disclosure of Interest:**

None Declared

